# Psychosis, Mood and Behavioral Disorders in Usher Syndrome: Review of the Literature

**Published:** 2015

**Authors:** Daniela Domanico, Serena Fragiotta, Alessandro Cutini, Pier Luigi Grenga, Enzo Maria Vingolo

**Affiliations:** Department of Medical–Surgical Sciences and Biotechnologies, U.O.S.D. Ophthalmology, Sapienza University of Rome, Terracina, Italy

**Keywords:** Usher Syndrome, Retinitis Pigmentosa, Schizophrenia, Psychosis, Hearing Loss

## Abstract

The aim of this review is to focus the current knowledge about mental and behavioral disorders in Usher syndrome. Previous studies described the presence of various mental disorders associated with Usher syndrome, suggesting possible mechanisms of association between these disorders. The most common manifestations are schizophrenia-like disorder and psychotic symptoms. Mood and behavioral disorders are rarely described, and often are associated with more complex cases in co-occurrence with other psychiatric disorders. Neuroimaging studies reported diffuse involvement of central nervous system (CNS) in Usher patients, suggesting a possible role of CNS damage in the pathogenesis of psychiatric manifestations. Genetic hypothesis and stress-related theories have also been proposed.

## INTRODUCTION

Usher syndrome (USH) is characterized by congenital sensorineural hearing loss, retinitis pigmentosa (RP), and sometimes, vestibular areflexia (1). The prevalence rate of USH is about 5–6 per 100,000 in northern Europe population and in the USA, and it represents the major cause of genetic deafness and blindness (1–4). Three clinical subtypes are distinguished on the basis of the severity of hearing loss, vestibular dysfunction and the age of onset of RP. Type I (USH1) is characterized by profound congenital deafness, prepubertal-onset RP, vestibular dysfunction. Usher syndrome type II (USH2) is characterized by congenital mild to severe hearing loss, adolescent-onset RP and no vestibular dysfunction. Usher syndrome type III (USH3) is characterized by rapidly progressive hearing loss. Age of onset of RP and the degree of vestibular dysfunction are variable (5–6).

Previous studies reported the presence of various mental disorders associated with Usher syndrome, suggesting mechanism of association between Usher syndrome and mental and behavioral disorder (7–9). Despite these findings, to date the literature offers fragmented information, mostly case reports, and few observational obsolete studies. In addition, there are no revisions that could help to focus the current knowledge about this association. This article reviews the current literature about the relationship of mental and behavioral disorders and Usher syndrome. The aims of this review include i) to report the prevalence and which type of disorders have been found in USH patients, ii) neuroradiological, clinical and molecular aspects that may predispose to mental and behavioral disorders and iii) current theories and the new hypotheses about relationships between these disorders and USH.

*Epidemiology*

There are conflicting data about the prevalence of psychotic disorders in USH patients. Hallgren (7) reported that psychosis was diagnosed in 26 of 114 persons (23% of cases) with Usher syndrome in a Swedish cohort. Nuutila (8) observed the prevalence of psychosis in 6 of 131 patients (4.5% of cases). He suggested that the data about psychosis prevalence in the Hallgren’s study was overestimate. Dammeyer (9) and Grøndahl and Mjøen (10) have confirmed these data, reporting schizophrenia in 1 of 26 patients (3.84%) and in 1 of 27 patients (3.70%), respectively. In addition, Dammeyer (9) reported that 23% of USH patients manifested mental or behavioral disorders, included schizophrenia, mild and severe mental retardation, atypical autism, and conduct disorder. The prevalence for schizophrenia is approximately 1.1% of the US population, (11) thus the prevalence of schizophrenia in Usher population is greater than US population. Nevertheless, the estimation of mental disability or psychosis in USH patients is difficult due to communication barriers.

**Figure 1:**
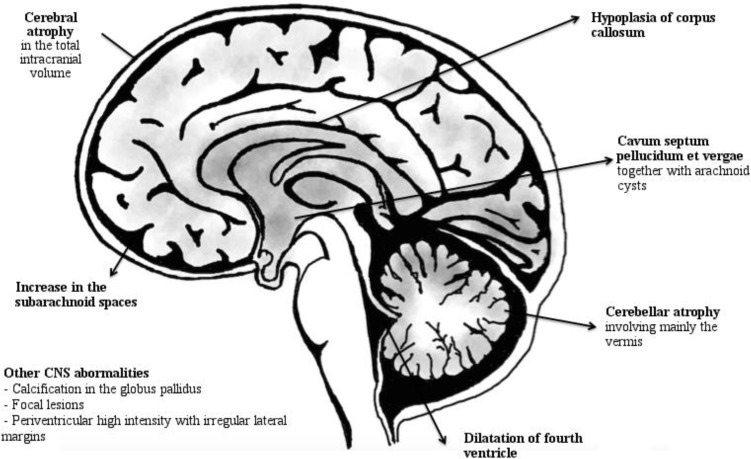
Summarized Neuroimaging Findings in USH Patients

*Usher Syndrome AND CNS Abnormalities (* Figure 1 *)*

Computed tomographic (CT) and magnetic resonance imaging (MRI) studies have shown various abnormalities of CNS. Cerebellar and cerebral atrophy, focal lesions, hypoplasia of corpus callosum and dilatation of fourth ventricle, decrease in intracranial volume with an increase in the size of the subarachnoid spaces, and arachnoid cyst together with cavum septi pellucidi et vergae. (12–16) The increase in the subarachnoid spaces seems to be related to the decrease in the total intracranial volume. These abnormalities are reported both in Usher type I and II, but USH type I patients are more severely affected with more prominent atrophy and cerebellar involvement (14). Tamayo et al. reported that 18 of 30 patients (60%) presented cerebellar abnormality at MRI of the brain.

The most common abnormal pattern (14 of 30 patients) was atrophy involving mainly the vermis. Although abnormal gait was very common in both USH type I (88.9%) and Type II (66.7%) patients, no correlation between cerebellar and gait abnormalities was found. According to the authors the pathogenesis of gait seems to be related to the loss of vestibular function in USH I and to cerebellar structural abnormalities in USH II (16). Positron emission tomography (PET) revealed a generalised reduction of cerebral blood flow and cerebral oxygen consumption in the cerebral cortex and subcortex, especially in the pallidum, but with relatively preservation of the basal ganglia (12). 

*Psychotic Disorders*

The association between Usher syndrome and psychiatric disorders is already known by many years. The most common manifestation reported is schizophrenia-like psychosis. (17–24)

There are a limited number of reports that offer a description about psychotic features of USH patients. Nevertheless, often the descriptions of auditory and visual hallucinations are only presumptive and based on the behavior of the subjects, due to poor communication skills of the patients. Sometimes the diagnosis of schizophrenia cannot be established with certainty; as well as psychometric evaluation cannot be performed in most cases. Table 1 reported case studies found in the literature regarding the association between Usher syndrome and psychiatric manifestations. We summarized the main mental and behavioral symptoms and the mental status.

In two case reports, the authors described the cases of two siblings with USH and psychotic symptoms (22,24). Furthermore, a positive family history of schizophrenia was reported in other two case reports (18,20). In most cases described, the authors reported a clinical picture resembling paranoid schizophrenia, none of these cases presented with catatonic symptoms.

Persecutory delusion was the most common form of delusions reported. In two cases were described religious delusions (17,19). The behavior was aggressive in the majority of cases. Moreover, patients were often severely anxious and irritable (see Table 1).

*Mood and Behaviour Disorders*

Rao et al. (25) first described a case of co-occurrence of bipolar disorder with psychotic symptoms and Usher syndrome. They have suggested that genes implicated in the development of Usher syndrome are present on the chromosomes 1,8,14, as well as chromosomes 8 and 14 are implicated in linkage studies of bipolar disorder. Praharaj et al. (26) also reported a case of mania associated with motor and language retardation in a patient affected by USH type II.

Tamayo et al. (16) reported abnormal mental status in 7 of 30 patients characterized by borderline mental retardation, depression or bipolar affective disorder. These disorders were observed in 16.7% of patients affected by USH I and in 33.3% with USH II.

Dammeyer reported that 6 of 26 children had mental and behavioral disorders: 1 with schizophrenia and mild mental retardation, 2 with atypical autism (1 of them with mild and the other with severe mental retardation), 1 with mild mental retardation and two with conduct disorder (9).

There were very few case studies that described clinical manifestations of mood and behavioral disorders in USH. Nevertheless, some cases reported the co-occurrence of attention deficit hyperactivity disorder, panic attack, anxiety, depression, and obsessive rituals with psychotic manifestation in USH patients. Moreover, in these patients learning disability is often suspected (17,21,23–24) (See Table 1).

**Table 1:** Summary of the Case Studies Found in the Literature Reporting Psychotic Symptoms in Patients With Usher Syndrome

	**USH type**	**Psychotic manifestations**	**Mood**	**Behaviour**	**Eat/Sleep**	**Mental Status**
**Mangotich and Misiaszek (1983)**	III (?)	Religious delusions, suicidal ideation	Agitation and anxiety		Sleep-	N.D.
**Hess-Röver et al. (1999)**	I	FH+ (1 PB schizophrenia), Visual and auditory hallucinations* (probably paranoid)	Anxious and distressed	Aggressive and bizarre	Sleep -	N.D., Intellectual disability Poor communication
**McDonald et al. (1998)**	II	Visual and auditory hallucinations Religious delusion (paranoid schizophrenia)				Borderline learning (IQ 74)
**Waldeck et al (2001)**	III	FH+ (sister) Capgras syndrome Paranoid	Angry, she refuses to accept the disease	Aggressive and self-injurious	Sleep-	N.D.
**Jumaian and Fergusson (2003)**	I	Visual and auditory hallucinations* (Schizophrenia unspecified)	Depressed, isolation		Anorexia	N.D. IQ- suspected;
**Wu and Chiu (2006)** **2 cases** **(Siblings)**	II II	Visual and auditory hallucinations* FH+ (sister) Auditory hallucinations and persecutory delusion*	Irritable	Aggressive		N.D. IQ -suspected; Elementary school N.D. IQ- suspected: High school
**Rijavec and Grubic (2009)**	III	Persecutory delusion, auditory hallucinations	Obsessive rituals, phobia, panic attack and anxiety, depression	Opposing behaviour, ADHD	Anorexia	N.D., Secondary school
**Domanico et al (2013)** **2 cases** **(Siblings)**	II II	FH+ (brother) Auditory hallucinations Visual and auditory hallucinations persecutory delusion (paranoid schizophrenia)	Irritable, isolation Irritable, isolation	Agitated ADHD Agitated and aggressive Panic like-attacks, anxiety and obsessive	Sleep- Sleep-	N.D., normal (Suspected) High school; N.D.; normal (suspected); Secondary expert technical school;

USH: Usher syndrome; ADHD: Attention deficit hyperactivity disorder; N.D.: not determinable; FH+: Positive familiar history; PB: parent’s brother;

* : suspected on the basis of the subject’s behaviour

## DISCUSSION

Although a substantial proportion of USH patients suffer from psychiatric symptoms, the etiopathogenesis remains unclear. Genetic hypothesis, brain damage and stress-related theories have been proposed. As reported above, two case reports described two siblings with both Usher and psychotic manifestations and other two cases of eight analysed reported the positive family history of schizophrenia (22,24,18,20). Based on these findings, we can speculate that there are families with a genetic predisposition or susceptibility to develop both USH and schizophrenia. Studies of families have been widely used to understand the relatives’ contributions of genetic and environmental factors upon risk for schizophrenia (27).

Two genetic loci associated to USH, 11q in type I and 5q in type II are also reported in schizophrenia, and it has been hypothesized that CNS defects may be among the pleiotropic effects of the USH gene (12,22). Although it has been hypothesized that one possible explanation for relationship between USH and psychotic illness was related to the closeness of genetic loci, which are more frequently inherited together because of linkage disequilibrium. Past studies reported that there was not overlap in chromosomal regions for USH and schizophrenia, without evidence of the loci on the same chromosome (18–20). Mutations in USH2A gene on chromosome 1q41 are the most common mutations (85% of all cases with USH2), the mutation of USH2A gene, 2299delG in exon 13 was found in two siblings affected by USH and psychotic manifestations (24, 28). In the schizophrenia, various genes are reported as possible causative genes, one of these recently discovered is DISC1 on chromosome 1q42.2 (27). This gene is on the same chromosome and close to the USH2A gene; therefore it could be interesting to further investigations. A second possible explanation is that Usher syndrome consists in a complex syndrome involving CNS diffusely. The involvement of CNS implies to consider an alternative scenario in which the encephalopathy can cause mental disorders. The psychiatric symptoms may be related to the diffuse cerebral involvement, this could explain why multiple or mixed mental and behavioral manifestations, such as hallucinations, delusional disorder, ADHD, depression, coexist in the same patient.

Several authors have proposed that CNS findings may be the consequence of the pleiotropic effect of the USH genes (14,16).

The stress-related theory was the first hypothesis proposed. This theory is supported by the fact that visual or auditory impairment is associated with a higher rate of depression, suicidal behavior, psychological stress and social handicap. Chronic stress may be related to progressive visual loss in a patient who has already experienced deafness, and has learned to use mostly the vision as the primary sense organ (17, 20). Indeed, patients with Usher syndrome and psychotic disorders often presented a mood characterized by anxiety, irritability, and aggressive behavior as the consequence of stress in a person already compromised by dual sensory impairments (18–20).

It is unclear to what degree the risk of psychosis may relate to progressive sensory impairment or to adaptation of the brain at changes in sensory input. It has been suggested that hallucinations in patients with visual loss are a consequence of abnormal release of central processing, as occurring in Charles Bonnet syndromes (17,19–20).).

## CONCLUSIONS

In conclusion, the pathogenesis of psychotic illness is probably multifactorial. Multiple genes and environmental factors, such as isolation, sensory deprivation, anxiety and stress-related disease may be involved. Similar to the pathogenesis of schizophrenia seems to be a strong genetic predisposition, in which stress-inducing factors, especially regarding sensorial deprivation, act on this susceptibility to trigger the disorder. The pleiotropic effect of the USH genes may cause the diffuse involvement of CNS. In addition, the diffuse abnormalities of CNS may be contributed to atypical or mixed psychiatric symptoms reported in USH patients. Further studies are necessary to confirm these hypotheses, but the association between USH and mental and behavioral disorders cannot be neglected. Therefore, an integrated approach with psychological support should be considered in these patients, to limit the psychological stress and consequently the development of mental disturbances.

## DISCLOSURE

The authors report no conflicts of interest in this work. 
